# *RAPIDviewer*: an *ImageJ* script for visualization and analysis of indexed electron diffraction patterns of cubic materials

**DOI:** 10.1107/S1600576726002086

**Published:** 2026-03-28

**Authors:** Thomas E. Weirich

**Affiliations:** a Gemeinschaftslabor für Elektronenmikroskopie (GFE) RWTH Aachen University Ahornstrasse 55 52074Aachen Germany; SLAC National Accelerator Laboratory, Menlo Park, USA

**Keywords:** electron diffraction, *ImageJ* macros, *RAPID* indexing, diffraction visualization, *RAPIDviewer*, cubic materials

## Abstract

*RAPIDviewer* is a newly developed *ImageJ* macro script that addresses the limitations of the indexing program *RAPID* (which enables the indexing of zone-axis electron diffraction patterns from cubic materials) by generating graphical overlays on the experimental patterns, including configurable spot and Kikuchi line drawings. Additional features include orientation sketches, inverse-pole-figure markers and an option for *VESTA* file export. Examples utilizing selected-area electron diffraction patterns from an austenitic steel, an M_23_C_6_ precipitate and an aluminium alloy illustrate the features of the program.

## Abstract 2

*RAPIDviewer* has been developed as an *ImageJ* macro that visualizes the indexing results of zone-axis-aligned electron diffraction patterns of cubic materials obtained with the program *RAPID*. The software facilitates the integration of crystallographic analysis within the *ImageJ* platform, offering a range of functionalities including configurable spot pattern overlays, Kikuchi line plotting, orientation plots and *VESTA* export.

## 1. Introduction

One of the earliest and most well established methods for determining crystal orientation and identifying crystalline phases is the geometric analysis of zone-axis-aligned electron diffraction patterns (Thomson & Cochrane, 1939 ▸; Andrews *et al.*, 1968 ▸; Burke, 2016 ▸). Recently, the macro script *RAPID* for the *ImageJ* platform was developed (Weirich, 2024*a* ▸), which allows instant indexing of calibrated and uncalibrated zone-axis-aligned electron diffraction patterns of cubic materials. However, as the results of the former are only provided in numerical form, users have relied on third-party programs to visualize the results until now. Therefore, a new *ImageJ* macro script called *RAPIDviewer* has been developed to carry out this task on the same platform. The functionality and features of this new program are described in the following sections and illustrated by examples.

## 2. Implementation, functionality and features of the *RAPIDviewer*

Like the indexing program *RAPID* (Weirich, 2024*a* ▸), the macro code of *RAPIDviewer* was developed using the freely available *FIJI* distribution of *ImageJ* (Schindelin *et al.*, 2012 ▸; Schneider *et al.*, 2012 ▸). The macro code is fully accessible and can easily be modified and extended to meet the specific needs of the users [see, for an introduction, Ferreira & Ehrenfeuchter (2022 ▸)].

**Figure 1:**
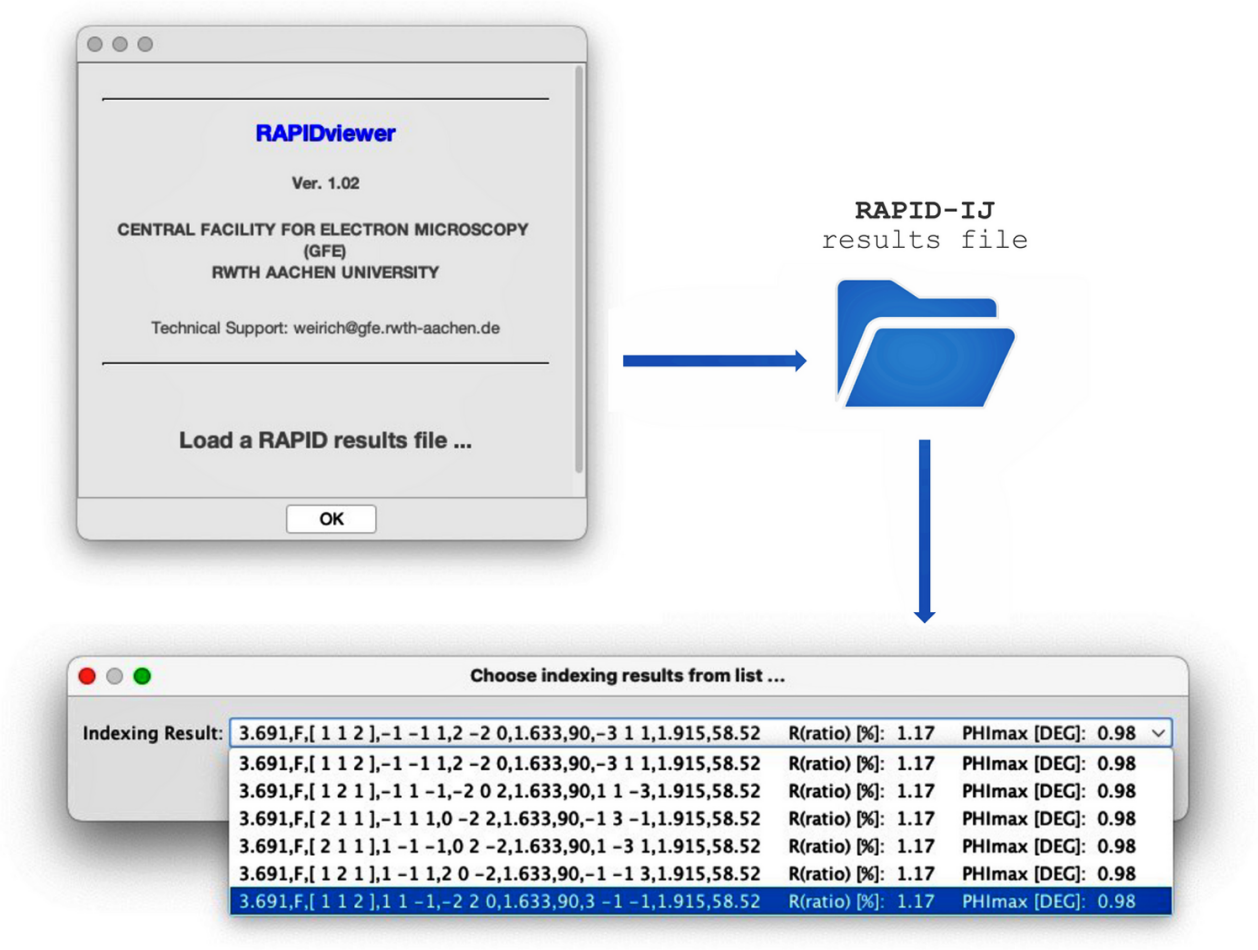
Workflow after starting the *RAPIDviewer* macro code in *ImageJ*. First, the user is asked to open a results file that has been generated by the *RAPID* indexing program (Weirich, 2024*a* ▸,*b* ▸). As the results file will usually contain several solutions for one particular electron diffraction pattern, a drop-down menu is used to select and load any of these solutions into the *RAPIDviewer* program. The diffraction-pattern image used for indexing with *RAPID* must be available in the same directory as the indexing results file.

Once *RAPIDviewer* has been started in *ImageJ*, the user is asked to open a *RAPID* results file from the hard drive. In the next step, the user can select any of the listed indexing solutions via a drop-down menu (Fig. 1 ▸).

**Figure 2:**
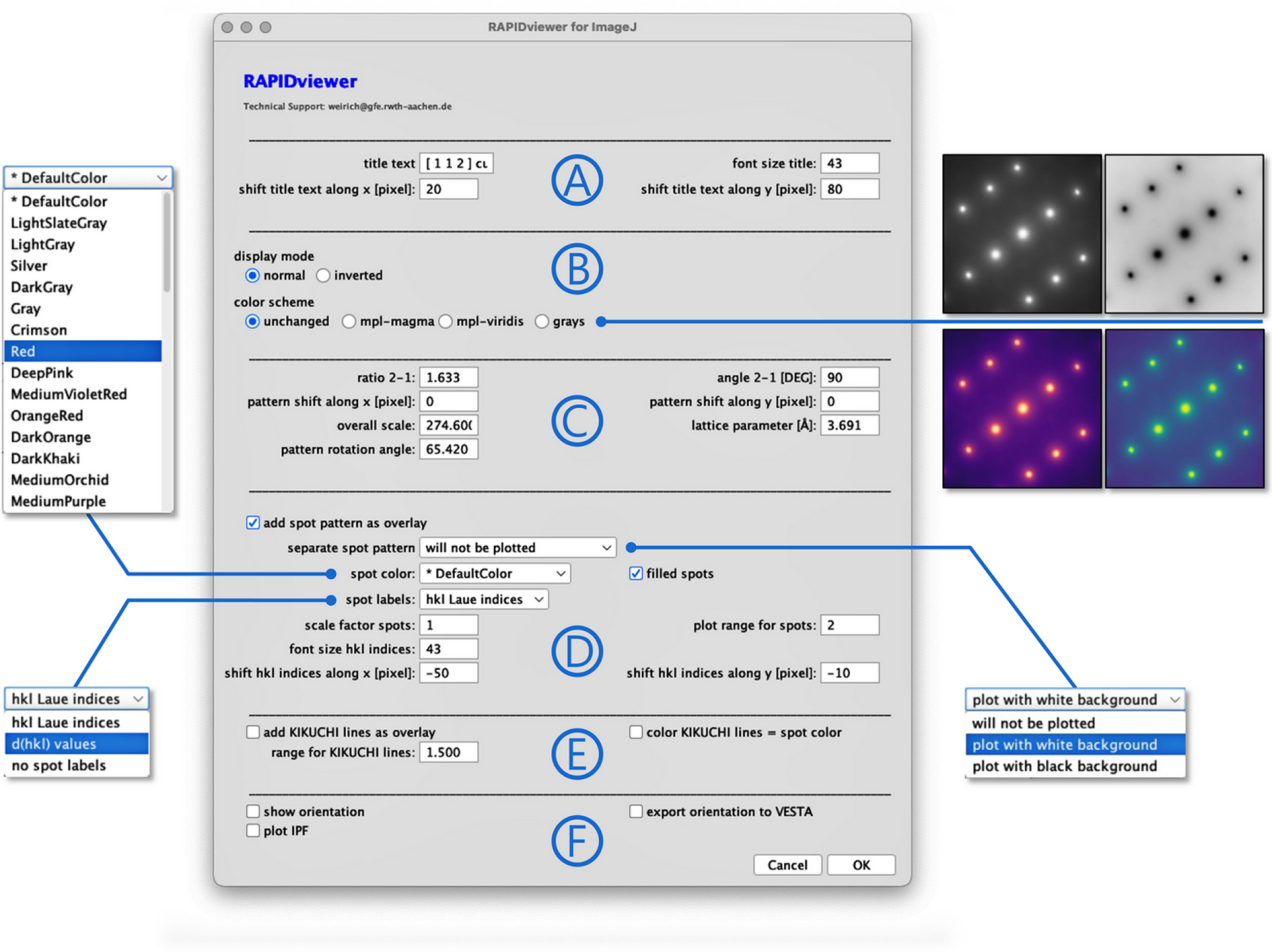
The user interface of *RAPIDviewer* consists of six main sections (A to F), which allow one to modify and adjust the graphical outputs of the program (for a detailed description of the options, see the main text). To operate the application, pressing ‘OK’ creates the desired output with the selected options and parameters. All graphical output is displayed on the monitor and is also automatically saved as a file to the hard disk. Subsequently the program returns to the main selection menu. Pressing ‘OK’ again repeats this cycle, whereby the saved files will be overwritten without notice. The program can be terminated at any time by pressing the ‘Cancel’ button.

When a particular indexing solution is loaded from the results file, a panel appears that allows the user to adjust and modify the appearance of the graphical outputs of the program. As shown in Fig. 2 ▸, this user interface consists of six main sections, A to F, which will be described below in detail.

**Figure 3:**
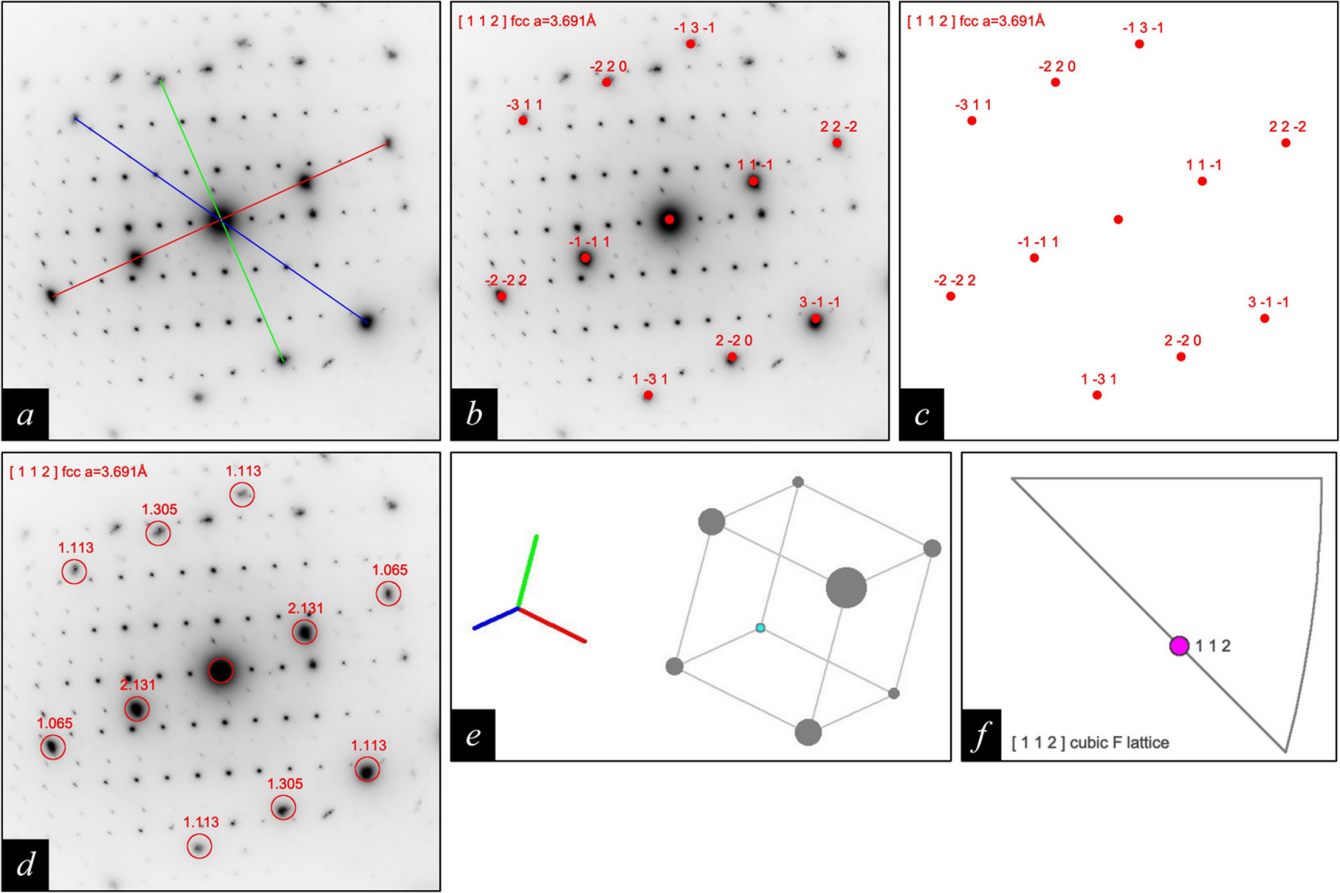
Processing the SAED pattern of an austenitic chrome–nickel–molybdenum steel in (*a*) with the program *RAPID* (Weirich, 2024*a* ▸,*b* ▸) led to the six indexing results shown in Fig. 1 ▸. The graphical outputs shown in (*b*) to (*f*) were obtained by using the last entry in the list with the *RAPIDviewer* program. As shown in (*b*), (*c*) and (*d*), the *RAPIDviewer* program allows one to index the diffraction spots either with *hkl* Laue indices or, if the lattice parameter of the cubic unit cell is known, with their corresponding *d*(*hkl*) values. For the latter, the ‘filled spots’ option was unchecked and ‘scale factor spots’ was set to 4. The figure in (*e*) shows the pseudo-3D representation for a primitive cubic unit cell in crystal space that has been determined by the program from the indexed [112] SAED pattern. Herein the red, green and blue lines refer to the cubic unit cell’s axes, *a*′, *a*′′ and *a*′′′, in that order, and the cyan point at the unit cell’s edge marks its origin. The IPF plot in (*f*) shows a marker for the [112] zone-axis direction within the standard stereographic triangle of the cubic system according to the common conventions (He, 2024 ▸).

All entries in section A of Fig. 2 ▸ are related to the title that is displayed in the top left corner of the experimental pattern [see, for example, Figs. 3(*a*), 3(*b*) and 3(*c*)]. These are the text content itself, the font size used, and the parameters for positioning the text along the *x* and *y* directions. The zone-axis indices *uvw* and the Bravais lattice type of the selected solution are used as the default text in each run. However, this annotation text can be replaced or completed by any other user-defined text.

In section B, the user can choose via radio buttons between different display modes for the processed electron diffraction pattern. Thus, the pattern can be displayed in its original colour scheme or with inverted colours. The latter can also be combined with the *ImageJ*-provided colour look-up tables ‘grays’, ‘mpl-magma’ and ‘mpl-viridis’.

Section C shows the ideal ratio and angle of the two base reflections of the selected indexing solution. These values, together with the values for the pattern scale (‘overall scale’) and pattern rotation, are used to draw a matching overlay on the experimental pattern. Aside from two more values for fine-tuning the shift of the calculated pattern along the *x* and *y* direction, a value for the average lattice parameter *a* determined earlier by *RAPID* is also displayed. The latter value is only used for labelling the diffraction spots with their corresponding *d*(*hkl*) spacings (see also the ‘spot labels’ option in section D). In this context, the *RAPID* results file for an uncalibrated pattern will provide the sum of the absolute indices of the two base reflections instead of a lattice parameter (Weirich, 2024*a* ▸). Therefore, it only becomes meaningful to label the spots in the diffraction pattern after this number has been replaced by a valid value for the lattice parameter.

Most of the options that affect the appearance of the output are found in section D. Here, it is possible to choose (via a checkbox) if the calculated pattern should be drawn as an overlay on the experimental image, and, from a dropdown list, it can be chosen whether a separate pattern is shown with a black or white background. Moreover, the colour of the diffraction spots and the type of spot labelling [*e.g.* as *hkl* Laue indices or *d*(*hkl*) values] can be selected from a dropdown list. Additionally, the user can select whether the spot markers are drawn as filled spots or circles and choose their size. Finally, it is possible to change the size of the corresponding *hkl* indices [or *d*(*hkl*) values], and to shift the position of the spot labels along the *x* and *y* axes if necessary. An important option in this section is the value for ‘plot range for spots’, since this value defines the radius within which the diffraction spots are drawn. The plot radius is obtained by multiplying the plot-range value and the length of the shortest reciprocal vector in the pattern.

Section E contains just a few options regarding Kikuchi lines. The drawing and colour of the lines can be selected using checkboxes, and the number of lines considered is defined using a range parameter that works similarly to the spot-range parameter in section D.

In section F, some additional outputs related to the determined zone axis can be selected. While ‘show orientation’ only provides a simple sketch of a primitive unit cell and its orientation in crystal space, ‘export orientation to VESTA’ generates an input file for the three-dimensional visualization system *VESTA* (Momma & Izumi, 2011 ▸), which can be used to generate publication-quality molecular drawings. Finally, the option to draw an inverse pole figure (IPF) can be selected, which marks the determined zone axis in the standard stereographic triangle of the cubic system (He, 2024 ▸).

## 3. Examples

To bypass the electron diffraction pattern indexing step with the base program *RAPID*, and to avoid duplicating the description of the indexing procedure, the following examples are the same as those used in the previous article (Weirich, 2024*a* ▸).

### 3.1. SAED pattern from an austenitic chrome–nickel–molybdenum steel

Fig. 3 ▸(*a*) shows a selected-area electron diffraction (SAED) pattern of an austenitic chrome–nickel–molybdenum steel, with the three lines used for indexing the steel matrix with the latest version of *RAPID* (Weirich, 2024*b* ▸). In accordance with the previous setting [see section 5.1 of Weirich (2024*a* ▸)], the corresponding solution for the [112] orientation was chosen from the *RAPID* results file (see Fig. 1 ▸). The overlay images in Figs. 3 ▸(*b*) to 3 ▸(*d*) were obtained by making the corresponding selections for spot colour and labels in section D of Fig. 2 ▸, and requesting a separate plot of the pattern with a white background. The orientation plots for the unit cell in crystal space [Fig. 3 ▸(*e*)] and the IPF [Fig. 3 ▸(*f*)] were obtained by selecting the respective checkboxes in section F of Fig. 2 ▸.

### 3.2. SAED pattern of M_23_C_6_ precipitate in austenitic chrome–nickel–molybdenum steel

**Figure 4:**
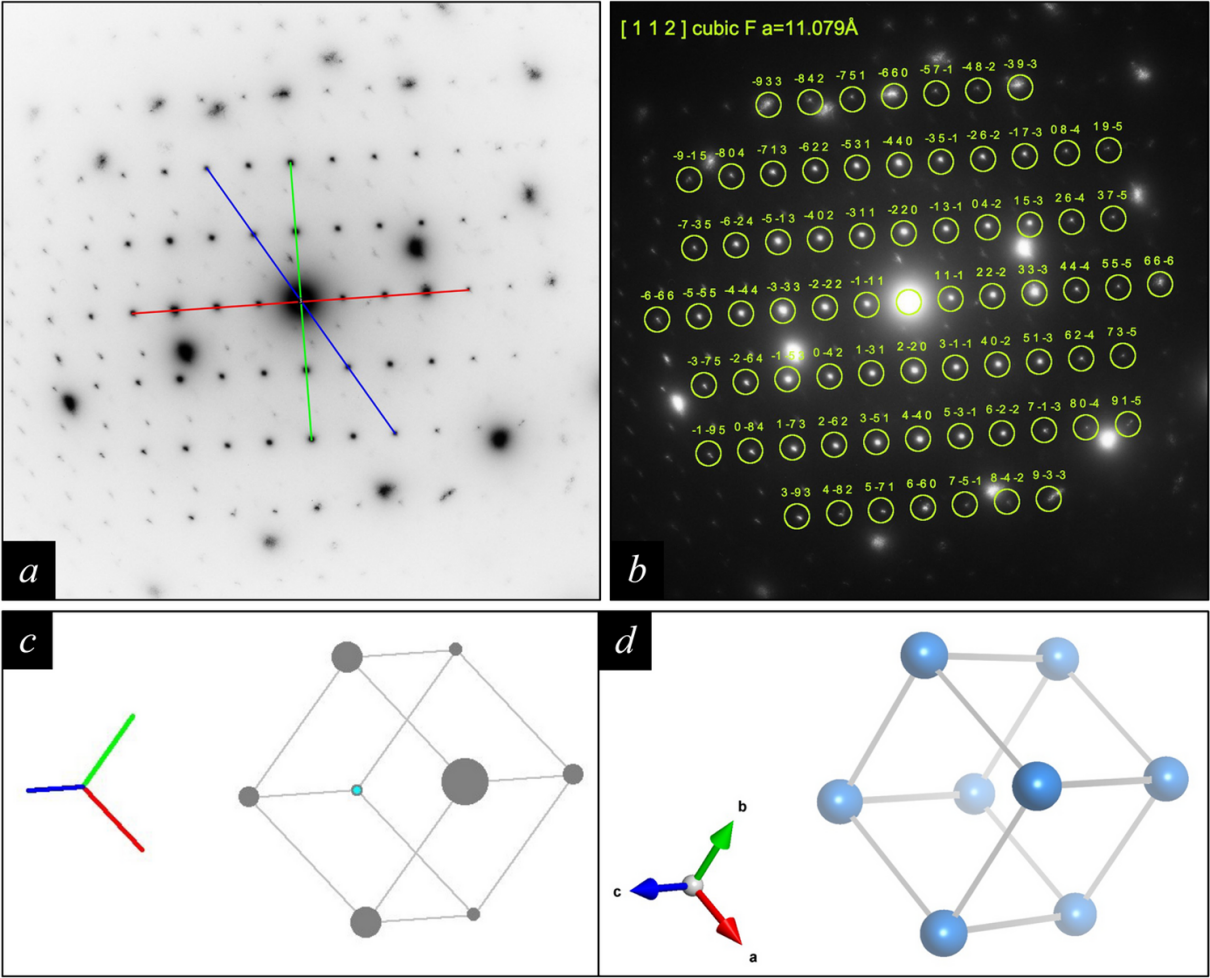
These series of images were obtained using the results from processing the partial pattern of an oriented incoherent precipitate with a D8_4_(M_23_C_6_) structure in (*a*) with *RAPID*. The pattern with overlay in (*b*) was obtained by choosing the inverted-pattern display mode in *RAPIDviewer* and using *hkl* Laue indices for the diffraction spots. Moreover, the spot markers were enlarged and the ‘filled spots’ checkbox was unselected. The orientation of the primitive unit cell in crystal space, corresponding to the identified [112] zone axis, is presented in (*c*). The 3D view of the unit-cell orientation shown in (*d*) is obtained when the output file generated by *RAPIDviewer* is opened in the program *VESTA* (Momma & Izumi, 2011 ▸).

Figs. 3 ▸(*a*) and 4 ▸(*a*) show, alongside the diffraction spots of the austenitic steel matrix, reflections of an oriented incoherent secondary phase with a D8_4_(M_23_C_6_) structure [for details see section 5.2 of Weirich (2024*a* ▸)]. As earlier for the steel matrix, the corresponding solution for the [112] orientation was selected from the *RAPID* results file, and this was used to produce the graphical outputs in Figs. 4 ▸(*b*) and 4 ▸(*c*). For comparison, Fig. 4 ▸(*d*) shows the corresponding unit-cell representation obtained when opening the *RAPIDviewer*-generated *VESTA* input file.

### 3.3. SAED Kikuchi pattern of alloy AlZn5Mg

**Figure 5:**
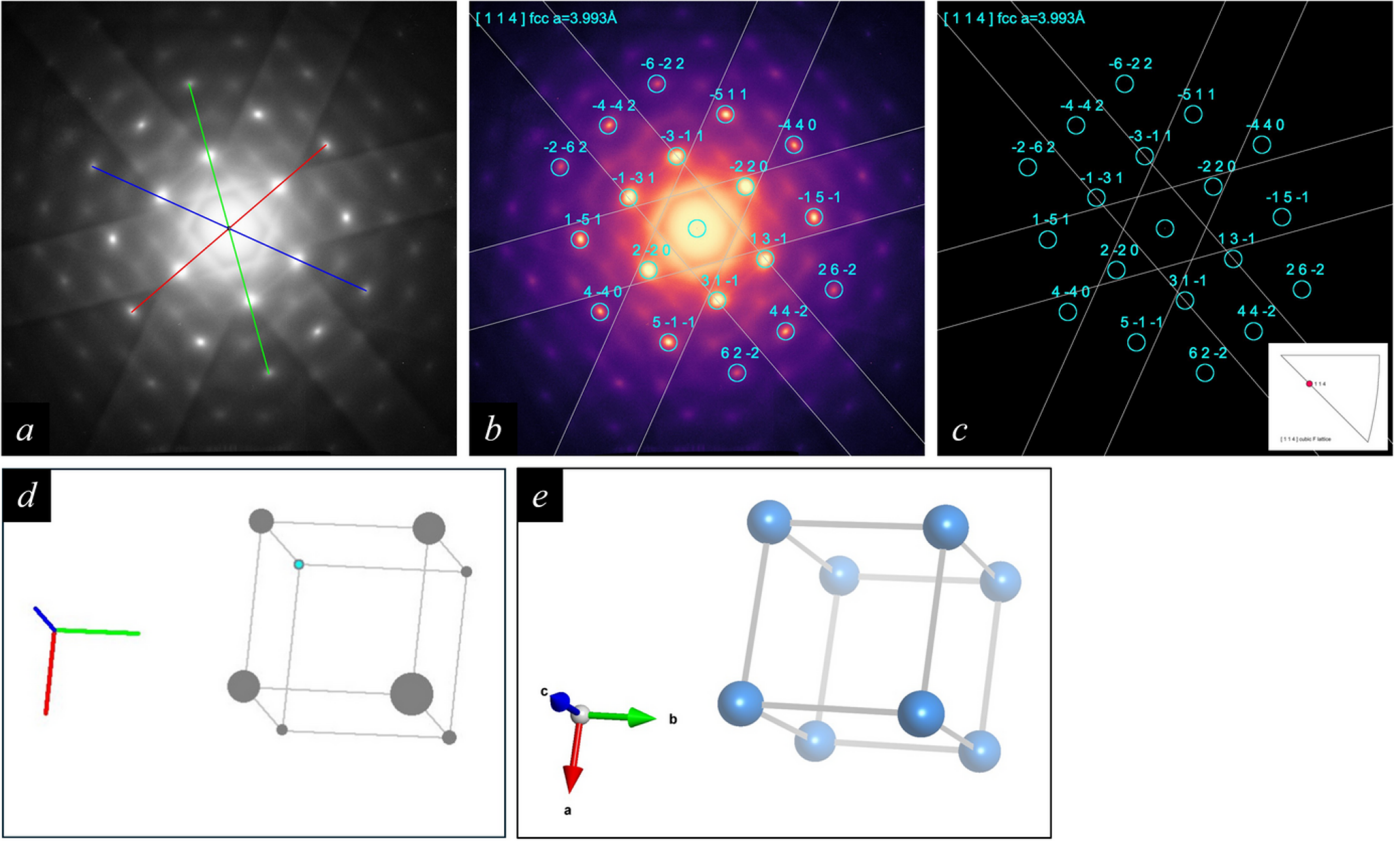
For the SAED pattern from aluminium alloy AlZn5Mg in (*a*), the colour scheme was changed to ‘mpl-magma’ and *hkl* Laue indices were chosen for labelling the diffraction spots in (*b*). Moreover, the option to add Kikuchi lines was selected and the diffraction spot markers were enlarged and drawn as unfilled circles. The same overlay, but without the experimental pattern and with a black background, is shown in (*c*). The corresponding unit-cell orientation along the [114] direction in crystal space is shown in (*d*). The 3D view obtained with the program *VESTA* (Momma & Izumi, 2011 ▸) when using the *RAPIDviewer*-generated input file for the same orientation is shown in (*e*).

A SAED pattern recorded from aluminium alloy AlZn5Mg along a zone axis was processed using the *RAPID* indexing program [Fig. 5 ▸(*a*)], and the solution corresponding to the previous result for the [114] orientation [see section 5.4 of Weirich (2024*a* ▸)] was selected for further processing with the program *RAPIDviewer*. The corresponding graphical output for a pattern with display mode ‘mpl-magma’, *hkl* Laue index labels and unfilled enlarged spot markers is shown in Fig. 5 ▸(*b*). In this case, the option that adds Kikuchi lines to the overlay has additionally been selected. A separate image that shows only the ideal spot pattern together with the Kikuchi lines on a black background is shown in Fig. 5 ▸(*c*). The latter can be requested via the corresponding drop-down menu in section D in Fig. 2 ▸.

## 4. Discussion and conclusions

The *ImageJ* macro script *RAPIDviewer* presented here integrates visualization capabilities directly into the workflow of the *RAPID* indexing program (Weirich, 2024*a* ▸) by eliminating the need for external plotting tools, and thus improves and simplifies the interpretation of the obtained indexing solutions. The program provides a large variety of options that allow users to tailor the overlays drawn on the experimental pattern. In addition, the program-generated orientation sketches, IPF plot and export option to *VESTA* (Momma & Izumi, 2011 ▸) allow one to link the diffraction-based orientation determination with the 3D structural interpretation. The latter is an important feature for the study and visualization of twins or for determining orientation relationships. Thus, with its flexible overlay options and orientation analysis features, *RAPIDviewer* offers a powerful and easy-to-use environment for the crystallographic interpretation of aligned electron diffraction patterns of cubic materials.

## Data Availability

The macro code of the program *RAPIDviewer* is published under GNU General Public License v3.0 or later at Zenodo (https://doi.org/10.5281/zenodo.19131217), which complies with the FAIR principles for research software (Barker *et al.*, 2022 ▸).
